# Atypical Atrial Flutter Unveiling Cor Triatriatum Sinister in a Stable Adult

**DOI:** 10.1016/j.jaccas.2026.109548

**Published:** 2026-07-31

**Authors:** Binay Panjiyar, Alejandro J. Herrera, John Hermann, Godfrey Tabowei, Abi Watts, Leela Lella

**Affiliations:** Texas Tech University Health Sciences Center, Permian Basin, Odessa, Texas, USA

**Keywords:** atrial flutter, cardiac magnetic resonance, congenital heart defect, echocardiography

## Abstract

**Background:**

Cor triatriatum sinister (CTS) is a rare congenital anomaly, infrequently identified in asymptomatic adults.

**Case Summary:**

A 64-year-old man presented with asymptomatic atrial flutter detected on routine electrocardiogram. Rate control was suboptimal, and because of unknown arrhythmia duration, transesophageal echocardiography (TEE) was performed before cardioversion. TEE excluded intracardiac thrombus and incidentally revealed a left atrial membrane. Cardiac magnetic resonance imaging confirmed nonobstructive CTS. The patient underwent successful TEE-guided electrical cardioversion and was discharged on anticoagulation.

**Discussion:**

CTS has been associated with atrial arrhythmia, although causality remains uncertain, particularly when arrhythmia originates from the right atrium. This case highlights the role of multimodality imaging in identifying incidental congenital anomalies and guiding safe management.

**Take-Home Messages:**

Careful atrial evaluation during TEE may reveal incidental CTS. Distinguishing associations from causation is essential for appropriate management.

## Visual Summary

**Figure:**
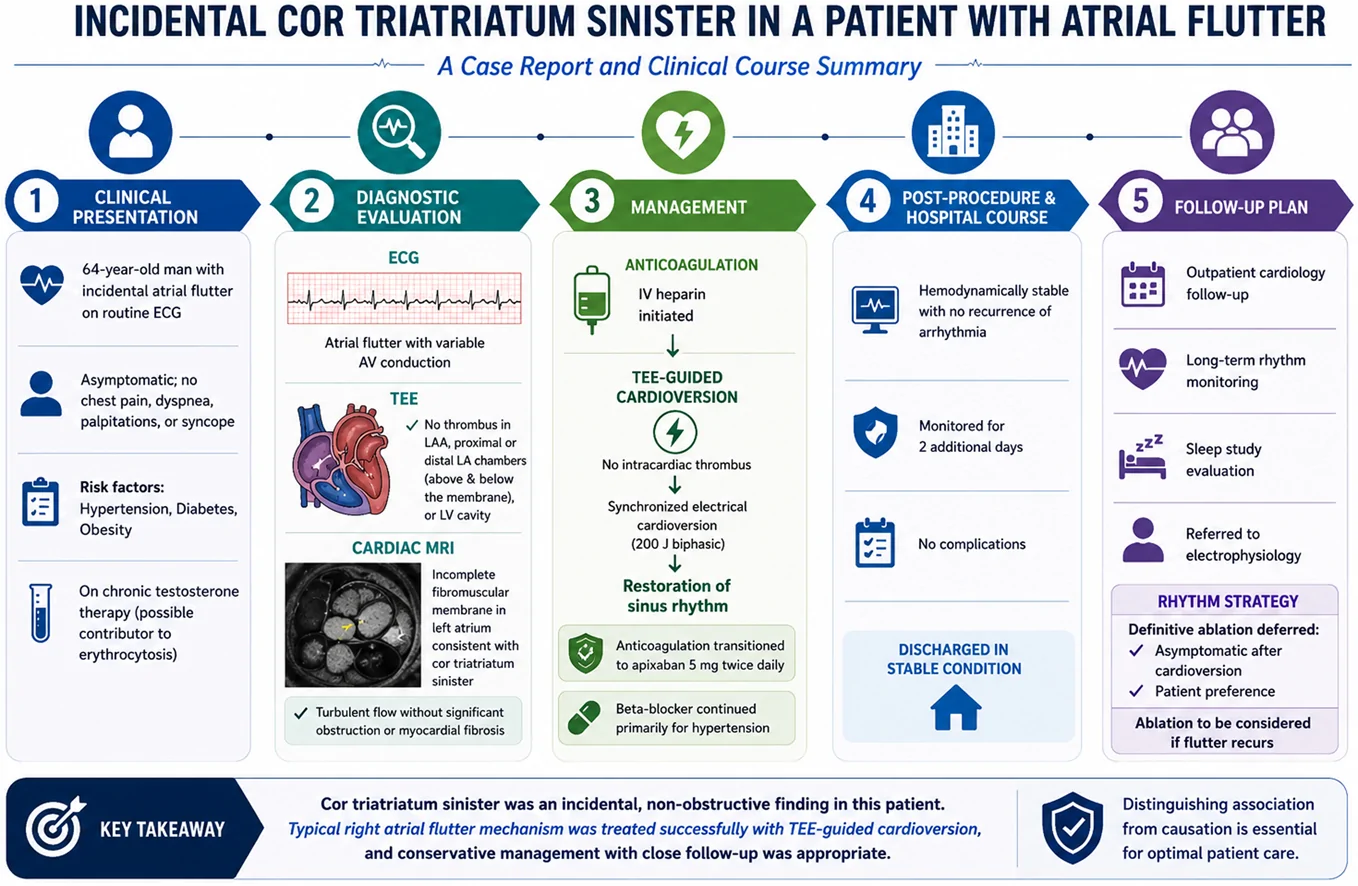

**Visual Summary:**
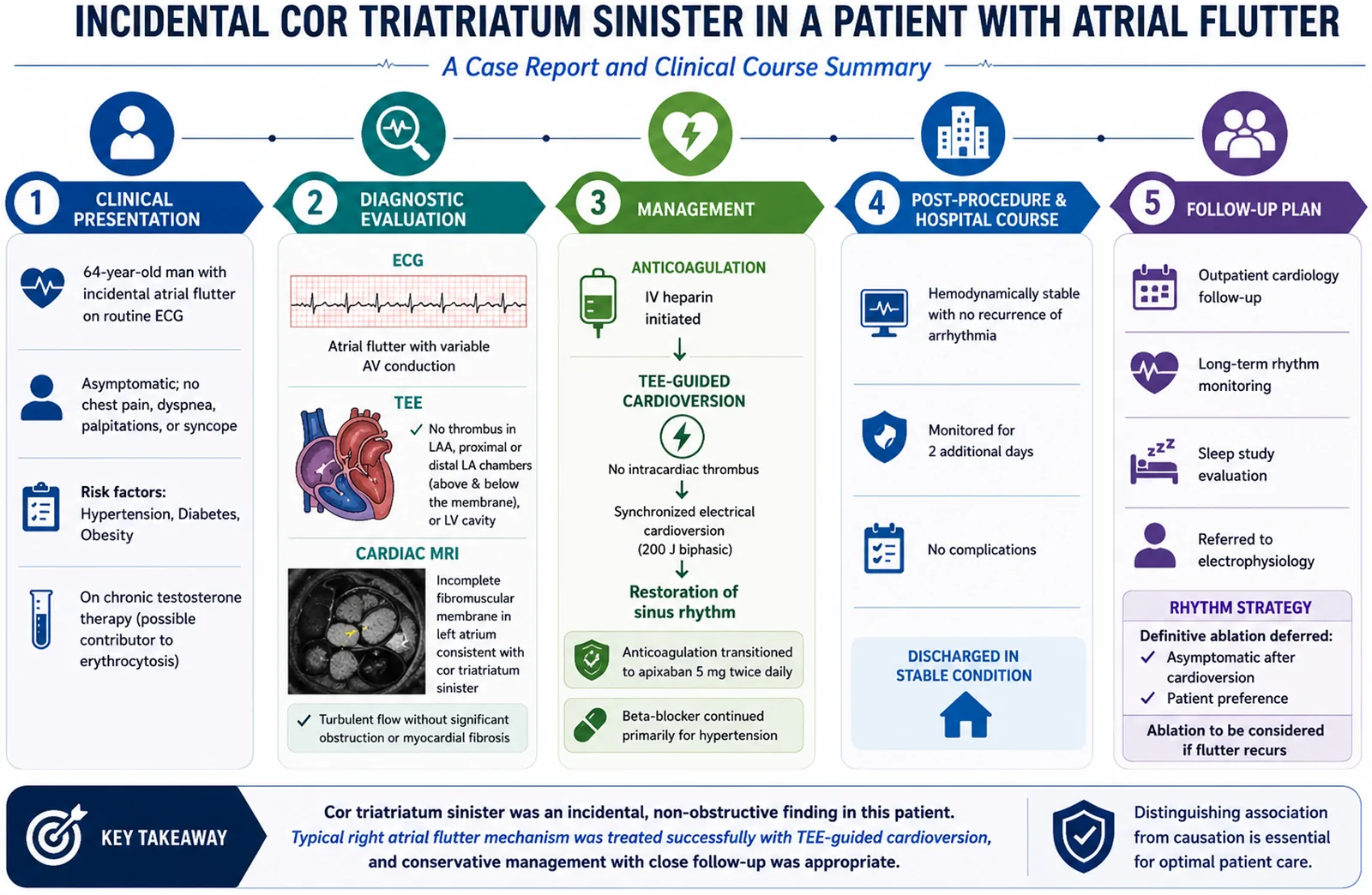
Typical Right Atrial Flutter was Managed Successfully With TEE-Guided Cardioversion and Conservative Follow-Up AV = atrioventricular; ECG = electrocardiogram; IV = intravenous; LA = left atrium; LAA = left atrial appendage; LV = left ventricle; MRI = magnetic resonance imaging; TEE = transesophageal echocardiography.

## History of Presentation

A 64-year-old man was referred to the emergency department after an abnormal electrocardiogram obtained during a routine primary care visit. The patient was asymptomatic at presentation and denied chest pain, dyspnea, palpitations, syncope, or constitutional symptoms. On arrival, he was hemodynamically stable but tachycardic. Electrocardiography demonstrated atrial flutter with variable atrioventricular block. He was admitted for telemetry monitoring and further evaluation.

**Take-Home Messages**

• Careful atrial evaluation during TEE may reveal incidental CTS.
• Distinguishing associations from causation is essential for appropriate management.

## Past Medical History

The patient had a medical history of well-controlled hypertension managed with losartan and atenolol, type 2 diabetes mellitus on sitagliptin/metformin and empagliflozin, hyperlipidemia on atorvastatin, and obesity with a body mass index of 30. He had no previous cardiac history, no smoking or drug use, and occasional alcohol use. There was no family history of congenital or structural heart disease.

## Physical Examination

On presentation, the patient was well-appearing and in no acute distress. Vital signs revealed a temperature of 36.6 °C, blood pressure of 143/85 mm Hg, respiratory rate of 18 breaths per minute, oxygen saturation of 98% on room air, and a heart rate of 124 beats/min on telemetry monitoring. Body mass index was 30 kg/m^2^.

Cardiovascular examination demonstrated a tachycardic irregular rhythm with normal S1 and S2 and no audible murmurs, rubs, or gallops. Peripheral pulses were symmetric with intact distal perfusion. Pulmonary examination revealed clear breath sounds bilaterally without wheezes, rales, or rhonchi, and there was no accessory muscle use or respiratory distress.

The abdomen was soft, nontender, and nondistended without guarding or palpable masses. Extremities showed no edema, erythema, or deformity. Neurologic examination demonstrated the patient to be alert and oriented, with normal speech and no focal motor or sensory deficits.

Skin examination was unremarkable, with warm, dry skin and no jaundice, petechiae, or rash. Head and neck examination was normal, with moist mucous membranes and no lymphadenopathy. Psychiatric evaluation demonstrated normal mood, affect, and linear thought processes.

## Differential Diagnosis

The initial differential diagnosis included atrial arrhythmias such as typical atrial flutter and atrial fibrillation. Secondary causes were considered, including metabolic and endocrine etiologies such as thyrotoxicosis. Acute coronary syndrome was included in the differential given the abnormal electrocardiogram, although it was considered less likely in the absence of symptoms and with normal cardiac biomarkers. Other considerations included pulmonary embolism and pericarditis. Structural heart disease, including previously unrecognized congenital anomalies, was also considered during further evaluation.

## Investigations

**Figure 1:**
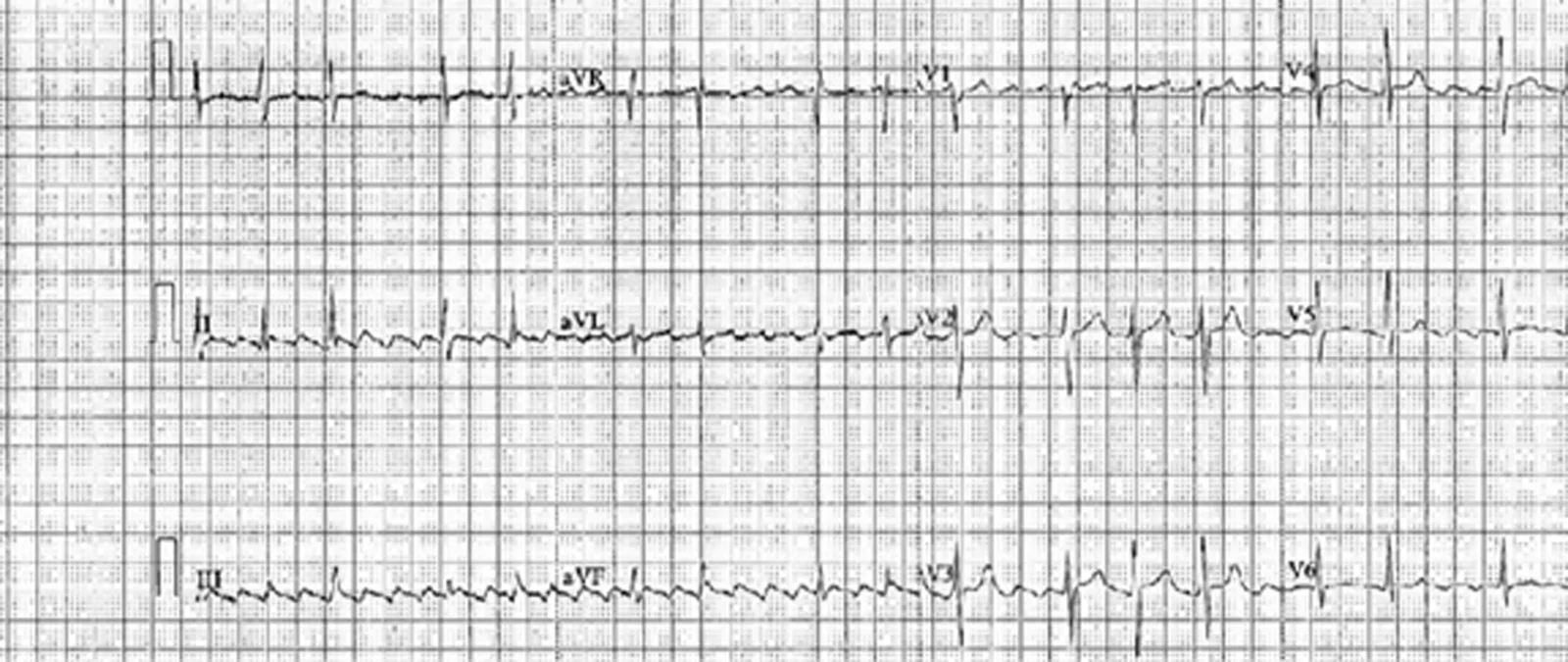
Electrocardiogram Demonstrating Typical Atrial Flutter With Sawtooth Flutter Waves and Variable Atrioventricular Conduction

Electrocardiography at presentation demonstrated atrial flutter, as shown in the Figure 1, with a ventricular response rate of approximately 93 beats/min and occasional premature ventricular complexes. The flutter-wave morphology was most consistent with a typical cavotricuspid isthmus-dependent right atrial flutter pattern. Chest radiography was unremarkable.

Laboratory evaluation, as shown in the Table 1, revealed a white blood cell count of 11.4 × 10^3^/μL, hemoglobin of 16.7 g/dL, serum glucose of 132 mg/dL, blood urea nitrogen of 11 mg/dL, and creatinine of 1.3 mg/dL. N-terminal pro–B-type natriuretic peptide was mildly elevated at 264 pg/mL and cardiac troponin I levels remained within normal limits. Thyroid function tests were normal. The patient was noted to be on chronic testosterone replacement therapy, which may have contributed to relative erythrocytosis.

**Table 1:** Laboratory Findings at Presentation

Category	Parameter	Value	Interpretation
Hematology	WBC	11.48 × 10^3^/μL	Mild leukocytosis
	Hemoglobin	16.7 g/dL	High-normal
	Hematocrit	51.6%	Upper range
	RBC	6.45 × 10^6^/μL	Elevated (likely testosterone-related)
	Platelets	226 × 10^3^/μL	Normal
Metabolic panel	Glucose	132 mg/dL	Mildly elevated
	Creatinine	1.3 mg/dL	Mildly elevated
	eGFR	56 mL/min/1.73 m^2^	Mildly reduced
	Sodium	137 mmol/L	Normal
	Potassium	4.6 mmol/L	Normal
Liver function	Total Bilirubin	1.7 mg/dL	Mildly elevated
	AST/ALT	38/32 U/L	Normal
Cardiac markers	Troponin I	<0.012 ng/mL	Normal
	NT-proBNP	264 pg/mL	Mildly elevated
Thyroid function	TSH	1.54 μIU/mL	Normal
	Free T4	1.04 ng/dL	Normal
Coagulation	INR	1.08	Normal
Other	Urine toxicology	Negative	No substance use

Laboratory evaluation demonstrated mild leukocytosis, relative erythrocytosis likely related to chronic testosterone therapy, mildly elevated N-terminal pro–B-type natriuretic peptide (NT-proBNP), and preserved thyroid and cardiac biomarker profiles without evidence of acute ischemia.

ALT = alanine aminotransferase; AST = aspartate aminotransferase; eGFR = estimated glomerular filtration rate; INR = international normalized ratio; RBC = red blood cell; TSH = thyroid stimulating hormone; WBC = white blood cell.

Given the unknown duration of atrial flutter and the planned rhythm-control strategy, transesophageal echocardiography (TEE) was performed before cardioversion to exclude intracardiac thrombus. TEE demonstrated a membranous septation within the left atrium without evidence of thrombus in the left atrial appendage, proximal or distal left atrial chambers (including above the membrane), or left ventricular cavity.

Cardiac magnetic resonance imaging (MRI) further characterized the anatomy, revealing an incomplete fibromuscular membrane extending from the posterior wall of the left atrium to the interatrial septum, consistent with cor triatriatum sinister (CTS). Turbulent intra-atrial flow was observed without evidence of significant obstruction, myocardial fibrosis, or impaired ventricular function. In the absence of intracardiac thrombus, the patient subsequently underwent TEE-guided synchronized electrical cardioversion.

## Management

The patient was admitted to telemetry for continuous cardiac monitoring and initiated on anticoagulation with intravenous heparin. Initial rate control was attempted with metoprolol tartrate; however, ventricular rate control remained suboptimal. Given the unknown duration of atrial flutter, TEE was performed before cardioversion to exclude intracardiac thrombus. TEE demonstrated no thrombus within the left atrium, including both proximal and distal chambers separated by the membrane, the left atrial appendage, or the left ventricular cavity.

Following thrombus exclusion, the patient underwent synchronized electrical cardioversion using a 200-J biphasic shock with successful restoration of sinus rhythm. Postprocedure, the patient remained hemodynamically stable without recurrence of atrial arrhythmia during hospitalization. Anticoagulation was transitioned to apixaban 5 mg twice daily, and beta-blocker therapy was continued primarily for hypertension management following restoration of sinus rhythm.

Electrophysiology services were not immediately available during the index hospitalization; therefore, outpatient electrophysiology follow-up was arranged. Given the patient's favorable clinical course, absence of recurrent symptoms, and maintenance of sinus rhythm after cardioversion, definitive invasive rhythm-control intervention was deferred at that time, with consideration for future catheter ablation should atrial flutter recur. The patient was discharged in stable condition with plans for outpatient cardiology follow-up, long-term rhythm monitoring, sleep study evaluation, and lifestyle optimization.

## Outcome and Follow-Up

The patient remained in sinus rhythm and asymptomatic at the time of discharge. Cardiac MRI confirmed the presence of CTS with no late gadolinium enhancement or myocardial scar. At 1-month follow-up, the patient remained asymptomatic with stable vital signs and no recurrence of atrial arrhythmia. He continued anticoagulation therapy, and beta-blocker therapy was maintained primarily for hypertension management. The patient was referred for outpatient electrophysiology evaluation, long-term rhythm monitoring, and lifestyle optimization, with consideration of catheter ablation should atrial flutter recur.

## Discussion With Literature Review

CTS is a rare congenital cardiac anomaly, accounting for approximately 0.1% to 0.4% of all congenital heart defects.^1^^,^^2^ It results from incomplete incorporation of the common pulmonary vein into the left atrium during embryogenesis, leading to the formation of a fibromuscular membrane that divides the left atrium into 2 chambers. The degree of functional obstruction depends on the size and number of fenestrations within the membrane. Severely restrictive membranes may mimic mitral stenosis physiology, whereas nonobstructive or fenestrated variants, as in this case, may remain clinically silent and be identified incidentally in adulthood.

In pediatric populations, CTS commonly presents with pulmonary venous hypertension, recurrent respiratory infections, dyspnea, or failure to thrive.^3^ In contrast, adult presentations are uncommon and are often discovered incidentally during evaluation for unrelated conditions, including atrial arrhythmias or embolic events.^4^^,^^5^ Increasing utilization of multimodality imaging, particularly TEE and cardiac MRI, has contributed to greater recognition of such congenital anomalies in asymptomatic adults.

In the present case, CTS was identified during evaluation of new-onset atrial flutter in a 64-year-old man. Although atrial fibrillation has more commonly been described in association with CTS, atrial flutter has also been reported, albeit less frequently.^6^ Importantly, the electrocardiographic morphology in this patient was most consistent with a typical cavotricuspid isthmus-dependent right atrial flutter mechanism rather than a left atrial arrhythmia. This distinction is clinically relevant, as it argues against the left atrial membrane serving as the direct arrhythmogenic substrate.

Although CTS has been associated with atrial arrhythmias in previous reports, causality cannot be definitively established in this case. The patient had multiple conventional risk factors for atrial arrhythmias, including advanced age, hypertension, diabetes mellitus, and obesity. Unlike conditions such as mitral stenosis, in which elevated left atrial pressure and atrial remodeling clearly predispose to arrhythmogenesis, the nonobstructive and fenestrated nature of the membrane in this patient makes a direct mechanistic relationship less convincing. Therefore, CTS in this context is best interpreted as an incidental structural finding rather than the primary cause of the atrial flutter.

Current guideline recommendations provide limited specific direction regarding management of CTS presenting with arrhythmias in adulthood. The 2020 European Society of Cardiology Guidelines for the Management of Adult Congenital Heart Disease recommend surgical membrane resection in symptomatic patients or those with significant obstruction.^7^^,^^8^ Similarly, the 2018 American Heart Association/American College of Crdiology Guidelines advocate individualized management based on symptom burden, hemodynamic impact, and anatomical findings.^9^ In asymptomatic patients without significant obstruction, conservative management is generally appropriate.

Management in this case was therefore directed toward the atrial flutter rather than the incidental structural abnormality. Initial rate control proved suboptimal, and given the unknown duration of arrhythmia, TEE-guided cardioversion was performed after exclusion of intracardiac thrombus. The patient underwent successful restoration of sinus rhythm and remained asymptomatic without recurrence during follow-up. Although cavotricuspid isthmus ablation would represent an effective definitive rhythm-control strategy for typical right atrial flutter, electrophysiology services were not immediately available during the index hospitalization. Outpatient electrophysiology follow-up was therefore arranged, and the patient elected to defer invasive rhythm-control intervention given his favorable clinical course after cardioversion, with future catheter ablation to be considered if recurrence occurs.

Advanced imaging played a central role in this case. TEE facilitated safe cardioversion through exclusion of thrombus while also identifying the intra-atrial membrane. Cardiac MRI further delineated the anatomy, confirmed the nonobstructive nature of the membrane, and demonstrated preserved ventricular function without fibrosis.^10^^,^^11^ These findings supported a favorable prognosis and justified a noninvasive management strategy.

This case reinforces an important clinical principle: incidental structural abnormalities may be identified during evaluation of atrial arrhythmias, but careful distinction between association and causation remains essential. In patients undergoing TEE for arrhythmia evaluation, systematic assessment of atrial anatomy may reveal rare congenital variants such as CTS that require appropriate anatomical characterization but may not necessarily alter immediate arrhythmia management.

Finally, this case contributes to the growing body of literature describing incidental adult presentations of CTS and highlights the importance of individualized, physiology-driven management. Although surgical correction remains indicated in symptomatic or obstructive cases, conservative management may be appropriate in asymptomatic patients with nonobstructive anatomy, as demonstrated in this case.

## Funding Support and Author Disclosures

The authors have reported that they have no relationships relevant to the contents of this paper to disclose.

## Appendix

Video 1 Cine Cardiac Magnetic Resonance Imaging Demonstrating Membranous Septation of the Left Atrium With Turbulent Intra-atrial Flow But Without Significant Obstruction, Consistent With Nonobstructive Cor Triatriatum Sinister

Video 2 Cine Cardiac Magnetic Resonance Imaging Demonstrating a Thin Fibromuscular Membrane Traversing the Left Atrium, Extending From the Posterior Left Atrial Wall Toward the Interatrial Septum, Consistent With Nonobstructive Cor Triatriatum Sinister, Without Evidence of Significant Inflow Obstruction or Myocardial Fibrosis

Video 3 TEE Demonstrating Nonobstructive Cor Triatriatum Sinister Without Intracardiac Thrombus Transesophageal echocardiography demonstrating a nonobstructive fibromuscular membrane traversing the left atrium, consistent with cor triatriatum sinister, without evidence of left atrial appendage thrombus or significant flow obstruction.
